# A CORS-Based Differential Correction Approach for AIS Mobile Stations

**DOI:** 10.3390/s18113626

**Published:** 2018-10-25

**Authors:** Qing Hu, Linlin Xu, Xinyu Cheng

**Affiliations:** Information Science and Technology College, Dalian Maritime University, Dalian 116026, China; hq0518@dlmu.edu.cn (Q.H.); xinyucheng@dlmu.edu.cn (X.C.)

**Keywords:** automatic identification system (AIS), continuously operating reference station (CORS), differential global positioning system (DGPS), AIS mobile stations

## Abstract

In order to remedy the inadequacy of the sources of differential corrections in current automatic identification system (AIS) and to improve the positioning accuracy of AIS mobile stations using single-point positioning, a differential correction approach for AIS mobile stations based on the continuously operating reference station (CORS) network is proposed. In the approach, AIS server derives real-time pseudo-range differential corrections from each reference station in CORS network and generates the corrections for AIS mobile stations. Then AIS base stations transmit these differential corrections to mobile stations using broadcast or addressed binary messages for positioning. Load analysis and testing show that this approach can effectively meet the need for differential corrections for most AIS mobile stations under the condition that the occupancy rate of the AIS channel is less than 1% when using broadcast binary messages. In addition, since this method is based on the existing CORS network, it is straightforward to implement in engineering projects and does not require additional hardware upgrades to the existing differential global positioning system (DGPS) and AIS infrastructure.

## 1. Introduction

The automatic identification system (AIS) is a novel type of navigational aid composed of shore-based facilities and shipborne equipment [1]. An AIS mobile station is a shipborne device that can provide information, such as unique identification, position, course, and speed to other vessels’ watchstanding officers and maritime authorities to track and monitor vessel movements. Reliability of the AIS system is closely related to the positioning precision of AIS mobile stations. The higher the positioning accuracy input to the AIS system, the safer navigation of the ship will be. However, some of the AIS mobile stations do not have the DGPS module and the single-point positioning accuracy of these mobile stations is only around 10 m which cannot meet the needs for ships when in crowded waters. Therefore, scholars are increasingly concerned about how to improve the positioning accuracy of the AIS system [2].

Currently, most AIS base stations and mobile stations support the transmission of differential corrections. AIS mobile stations can use existing AIS data links to perform DGPS positioning in order to improve accuracy [3,4]. However, how to get a stable and reliable source of differential corrections and to generate the corrections for AIS mobile stations is a problem that has to be addressed. In research on road network positioning, Rohani et al. [5,6] put forward the dynamic base station DGPS (DDGPS) concept, which uses the mobile user as the dynamic reference station to carry out differential corrections transmission to achieve cooperative navigation. However, it is not applicable to ships because it is built on the premise that accurate positioning is attainable. The high-precision network real-time kinematic (RTK) positioning model for vehicles in a downtown region proposed by Kim et al. [7] is not compatible with existing AIS. In terms of ship positioning, Zhang Peng et al. [8] made the proposal that GPS reference stations should be exclusively established for AIS base stations in order to facilitate differential corrections transmission. Even though it is an effective source for differential corrections, infrastructure costs would be extraordinarily high if the aim is for full coastal coverage. Therefore, this paper focuses on how to provide differential service for a great number of AIS mobile stations using existing systems.

Since technology in the field of geodetic survey is rapidly developing, the continuously operating reference station (CORS) network is used for high-precision network RTK positioning [9,10,11]. In addition, there are about 20,000 CORSs worldwide are available. So we choose CORS network as the source of differential corrections. We derive differential corrections to the code pseudo-range measurements from CORS network, because only the pseudo-range differential corrections are supported by the parameters of AIS messages. To be specific, this paper uses differential corrections of three reference stations to generate differential corrections for AIS mobile stations. Then AIS base stations transmit these differential corrections to mobile stations using broadcast or addressed binary messages. Based on testing results, it was found that using the method proposed in this article the AIS mobile station’s static and dynamic positioning accuracy reaches 1.06 m (2DRMS) and 2.54 m (2DRMS), respectively, which are far better than the existing single-point positioning methods. In addition, since it uses the existing CORS network with AIS base stations, spending is greatly reduced, and so the method is economically feasible.

The rest of the paper is organized as follows. Section 2 introduces the conceptual model of the CORS-based differential correction approach for AIS mobile stations. Section 3 describes the way to generate and transmit differential corrections to AIS mobile stations. Section 4 analyzes the approach’s impact on the AIS channel load. Section 5 provides the measurements and analysis, and is followed by the conclusions.

## 2. A Conceptual Model of the CORS-Based Differential Correction Approach for AIS Mobile Stations

**Figure 1:**
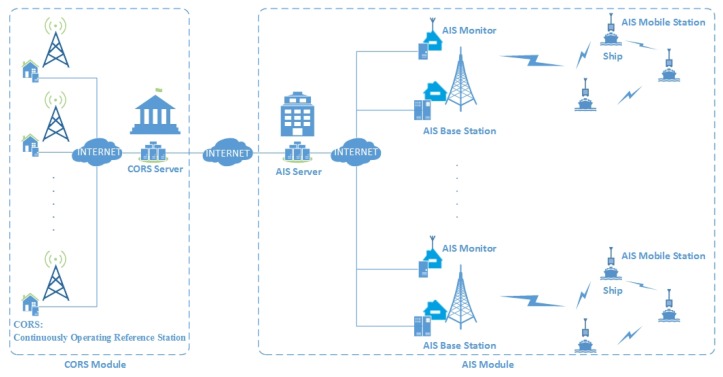
The conceptual model of the differential correction approach for AIS mobile stations.

The conceptual model of the differential correction approach for AIS mobile stations comprises two parts: the CORS module and the AIS module, as shown in Figure 1.

The CORS module includes the CORS server and CORSs. Each reference station has a GPS receiver and a computer located at the site [12]. The GPS receiver tracks and captures satellite signals including code pseudo-ranges and carrier phases around the clock. The main function of the computer is to record the observation data. The CORS server is a high-performance computer which is used to control and monitor all the CORSs remotely. CORSs are connected to the CORS server over the Internet and transfer the GPS data to this server. Another function of the CORS server is to distribute GPS data online. So we can get them through specific software interfaces. The CORS network can provide RTK differential data and pseudo-range differential corrections. But we only obtain pseudo-range differential corrections from the CORS server since RTK data cannot be used by AIS.

The AIS module includes the AIS server, AIS base stations, AIS monitors, and AIS mobile stations. AIS server has many functions, including the following: Communication: AIS server obtains differential corrections from CORS server and exchanges data with AIS base stations and AIS monitors. Calculation: AIS server generates differential corrections for AIS mobile stations. Control: AIS server is in charge of the remote controlling and monitoring of the AIS base stations. The AIS base stations transmit the differential corrections through VHF data link. The AIS mobile stations receive these data and correct pseudoranges. The AIS monitor is responsible for monitoring the accuracy of the corrections broadcast by the AIS base stations. If the data is incorrect, AIS server will inform the AIS base stations to stop broadcasting and detect the problem.

## 3. A Differential Correction Approach for AIS Mobile Stations

In order to provide DGPS service for AIS mobile stations which do not have a DGPS receiver, the differential corrections must be transmit using AIS messages. Since either broadcast or addressed binary messages can be sent in AIS using time division multiple access (TDMA) techniques, the differential correction approach is divided into two categories known as the differential correction approach for AIS mobile stations using broadcast binary messages and the differential correction approach for AIS mobile stations using addressed binary messages.

### 3.1. The Differential Correction Approach for AIS Mobile Stations Using Broadcast Binary Messages

**Figure 2:**
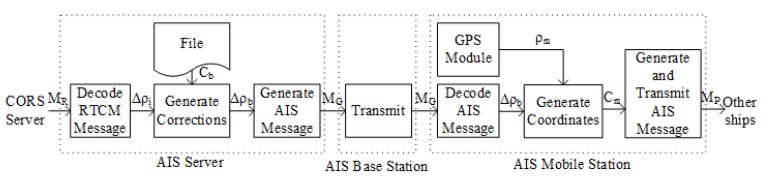
Block Diagram of the differential correction approach using broadcast binary messages.

The AIS message type 17, global navigation-satellite system broadcast binary message, can be used by a base station to broadcast differential corrections for GPS. The differential correction data section is in accordance with Recommendation ITU-R M.823 and organized as listed in Table 1. In addition, the block diagram of the differential correction approach for AIS mobile stations using broadcast binary messages is shown in Figure 2. The descriptions of related notations are given in Table 2.

The RTCM [13] messages of each reference station are acquired from the CORS server through a TCP port and decoded by the AIS server to obtain differential corrections. Available differential corrections are obtained by determining whether the epochs are the same. The differential corrections are generated according to the approximate coordinates of the AIS base station using the method proposed in Section 3.3. The AIS base station is permanently fixed to the same location, and, as a result, its location is known with a high degree of certainty. AIS server encodes the differential corrections of the AIS base station into global navigation-satellite system broadcast binary messages. The AIS base station broadcasts the messages to AIS mobile stations via AIS channel (161.975 MH, 162.025 MH). The AIS mobile stations receive the differential corrections and use them to correct pseudoranges. The positions are broadcast using AIS position reports to assist officials to identify each vessel’s position, movement, and create a picture of it in real time.

This approach generates and transmits the differential corrections of the AIS base station to AIS mobile stations based on the following factors: First, the AIS base stations is usually located near the port, while the reference stations in the CORS network is farther away from it. Secondly, the closer to the port, the greater the density of the vessel and the higher the positioning accuracy required by AIS mobile stations. Furthermore, the AIS mobile stations receiving the signals transmitted by an AIS base station must be within a certain distance from the AIS base station. In short, the AIS mobile stations are closely connected with the AIS base station and can use the differential corrections of them to increase the accuracy of GPS locations. However, the longer the distance between the AIS mobile station and the AIS base station, the weaker the correlation. In order to eliminate the influence of this factor, alternative, the differential correction approach for AIS mobile stations using addressed binary messages is proposed.

### 3.2. The Differential Correction Approach for AIS Mobile Stations Using Addressed Binary Messages

The AIS message type 6, addressed binary message, can be used by a base station to transmit differential corrections to a destination station. The parameters of an addressed binary message are given in Table 3.

**Figure 3:**
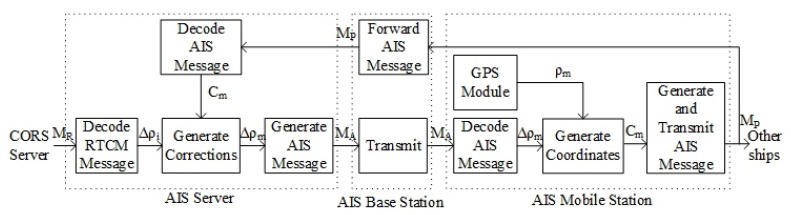
Block Diagram of the differential correction approach using addressed binary messages.

Each AIS base station and mobile station has a unique Maritime Mobile Service Identity (MMSI) which is a series of nine digits. In this approach the global navigation-satellite system broadcast binary messages (type 17) are transmitted as the binary data section of addressed binary messages (type 6). The block diagram of the differential correction approach for AIS mobile stations using addressed binary messages is shown in Figure 3. The descriptions of related notations are given in Table 2.

Most of the notations are same as the approach using broadcast messages except $\Delta \rho_{m}$ which means the differential corrections of the AIS mobile station and $M_{A}$ which means addressed binary messages. The main functions of the AIS server are along the same lines as the approach using broadcast messages. But the differential corrections are generated according to the approximate coordinates of the AIS mobile station. In contrast with the approach using broadcast messages, the positioning accuracy of the AIS mobile station in this approach is not related to the distance between the AIS base station and the mobile station. Therefore, for AIS mobile station far from the AIS base station, the positioning accuracy is improved.

### 3.3. A Method for Generation of Corrections to the Code Measurements in a Network of Reference Stations

With increasing distances between mobile stations and reference stations, the positioning accuracy of a mobile station in the traditional pseudo-range differential positioning model is becoming less. In order to model the distance-dependent biases between the reference stations and the user receiver with the support of a reference station network, several techniques have been developed. They include the linear combination model, distance-based linear interpolation method, linear interpolation method, lower-order surface model, and least squares collocation [14,15,16,17]. All of them use a n−1 independent error vector generated from a *n* reference station network to interpolate the distance-dependent biases for the user station location:

(1) $${\hat{V}}_{u}=\vec{\alpha }\cdot \vec{V}=\alpha_{1}V_{1n}+\alpha_{2}V_{2n}+\cdots +\alpha_{n-1}V_{n-1,n}$$

In this paper the linear combination model proposed by Han [18,19] in 1996 was selected to model the spatially correlated biases: orbit bias, residual ionospheric bias and residual tropospheric bias. The single-differenced pseudo-range can be written as:

(2) $$\begin{array}{c} \sum_{i=1}^{n}\alpha_{i}\cdot \Delta R_{i}=\sum_{i=1}^{n}\alpha_{i}\cdot \Delta \rho_{i}+\sum_{i=1}^{n}\alpha_{i}\cdot \Delta d\rho_{i}-c\cdot \sum_{i=1}^{n}\alpha_{i}\cdot \Delta dT_{i}\\ -\sum_{i=1}^{n}\alpha_{i}\cdot \Delta d_{ioni}+\sum_{i=1}^{n}\alpha_{i}\cdot \Delta d_{tropi}+\varepsilon_{\sum_{i=1}^{n}\alpha_{i}\cdot \Delta R_{i}} \end{array}$$

where $\Delta {\left(\cdot \right)}_{i}={\left(\cdot \right)}_{u}-{\left(\cdot \right)}_{i}$; i indicates the reference station i, and u the user station; ρ: the geometrical distance between the reference station and satellite; dρ: the effect of ephemeris errors, including S/A effects; ΔdT: the receiver clock error with respect to GPS time; $\Delta d_{ion}$: the ionospheric delay; $\Delta d_{trop}$: the tropospheric delay; ε: the carrier noise; c: the speed of light. A set of parameters $\alpha_{i}$ can be determined, based on the following conditions:

(3) $$\left\{\begin{array}{c} \sum_{i=1}^{n}\alpha_{i}=1\\ \sum_{i=1}^{n}\alpha_{i}\left(X_{u}-X_{i}\right)=0\\ \sum_{i=1}^{n}\alpha_{i}^{2}=Min \end{array}\right.$$

where ${\vec{X}}_{u}$ and ${\vec{X}}_{i}$ are the position vector in the Gauss plane coordinate system. It can be seen that all the coefficients can be calculated without using any actual measurements, and are constant if the user receiver is not in motion. Normally, three or more reference stations are required to meet the needs [20]. As a result, in this paper three reference stations a, b, and c are selected as the basic framework. The user u is the AIS base station in the differential correction approach for AIS mobile stations using broadcast binary messages. Accordingly, it is the AIS mobile station in the differential correction approach for AIS mobile stations using addressed binary messages.

## 4. Channel Load Analysis of the Differential Correction Approach for AIS Mobile Stations

The channel capacity resources of the AIS system are limited. In terms of use, the influence of the approach on the channel load rate of the AIS system should be taken into account. Through a simulation analysis, we selected a reasonable broadcast interval to provide differential corrections without affecting the effectiveness of the AIS system.

### 4.1. Channel Load Analysis of the Differential Correction Approach for AIS Mobile Stations Using Broadcast Binary Messages

In the AIS system, there are 2250 time slots per minute and two channels for broadcast. The total capacity of the AIS system is $S_{c}$, where:

(4) $$S_{c}=2\times n_{0}$$

where $n_{0}$ denotes 2250 time slots and the channel load P can be expressed as

(5) $$P=\frac{60/t_{i}\times n_{slot}}{S_{c}}$$

where $t_{i}$ is the interval of the AIS message type 17 broadcast by the AIS base station and $n_{slot}$ is the number of time slots occupied by the message.

**Figure 4:**
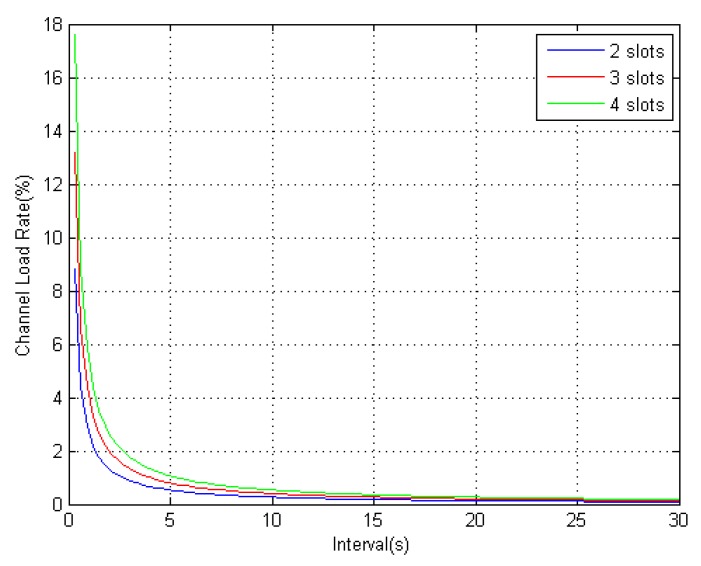
Channel load rate when using broadcast binary messages.

According to the regulation of AIS message type 17, when the number of transmitted satellites is not more than 10, the differential corrections broadcast by the AIS base station occupies two time slots. When the number of transmitted satellites is in the range to 19, the differential corrections occupies three time slots. The relationship between the AIS channel load rate and the differential message broadcast interval is shown in Figure 4.

From Figure 4, it can be seen that, when the broadcast interval is greater than 5 s, the AIS channel load rates with different time slots are less than 1%. The impact on the AIS system can be ignored. According to the existing theory, the differential corrections are time-dependent when there is SA interference. The accuracy of DGPS location is determined by the broadcast interval of the differential corrections. However, when there is no SA interference, the correlation between differential corrections and time is greatly reduced. Therefore, under the condition that the AIS channel occupancy rate is less than 1%, setting the broadcast interval at 5 s optimizes performance.

### 4.2. Channel Load Analysis for the Differential Correction Approach for AIS Mobile Stations Using Addressed Binary Messages

Compared with the differential correction approach for AIS mobile stations using broadcast binary messages, implementation of using addressed binary messages is more complex. It requires AIS addressed binary message to broadcast global navigation-satellite system broadcast binary messages, and the total number of binary bits transmitted must satisfy the following relationship:

(6) $$B_{sum}=B_{ais17-header}+B_{dgps-header}+B_{\mathrm{sv}}$$

where $B_{sum}$ indicates the number of bits that need to be transmitted in AIS message type 6. $B_{ais17-header}$ indicates the header of AIS message type 17, which is the fixed 80 bits. $B_{dgps-header}$ is the header of DGPS data, which is the fixed 40 bits. $B_{\mathrm{sv}}$ represents the number of bits of differential corrections. For each satellite, 24 bits are required. Therefore, when there are no more than 15 observation satellites, three time slots are needed for the transmission of AIS message type 6. When there are between 16 and up to 25 observation satellites, four time slots are required. Assuming that the AIS base station sends an AIS differential message to $n_{\mathrm{ship}}$ ships, the channel load rate P can be expressed as the CORS system can provide different forms of observation data according to the requirements of the mobile station. These include RTK differential data and pseudo-range differential data. Since the AIS mobile station employs DGPS for positioning, pseudo-range differential corrections from the CORS system are obtained in real time for calculation.

(7) $$P=\frac{n_{\mathrm{ship}}\times 60/t_{i}\times n_{slot}}{S_{c}}$$

**Figure 5:**
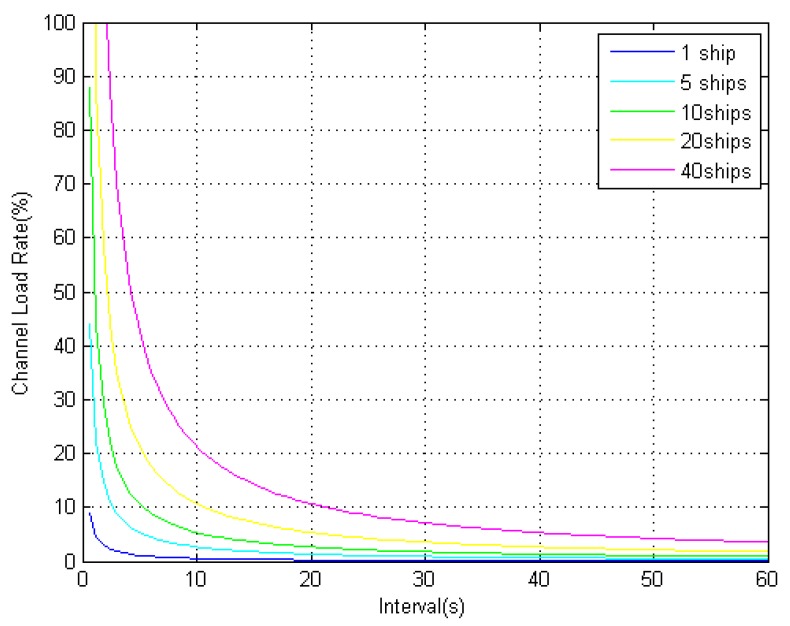
The channel load rate when using addressed binary messages.

When there are no more than 25 observation satellites, the relationship between the AIS channel load and the differential message addressing broadcast interval is as shown in Figure 5.

From Figure 5, it can be seen that the channel load rate is related to the broadcast interval and the number of ships. As the number of ships increases, the channel load increases linearly. If the research adopts the 15 s broadcast interval suggested by IALA, differential corrections can be simultaneously distributed to 10 mobile stations as long as the channel occupancy rate reaches 5%. Clearly too many ships will have a significant impact on the performance of the AIS system. Therefore, this model is only suitable for providing differential corrections for vessels at high risk or for low density shipping in inland rivers.

## 5. Experiments on System Accuracy

The differential correction approach for AIS mobile stations using broadcast binary messages was employed to conduct tests according to the channel load analysis in Section 4. A quantitative analysis of positioning accuracy is shown in the following.

### 5.1. Experimental Platform

**Figure 6:**
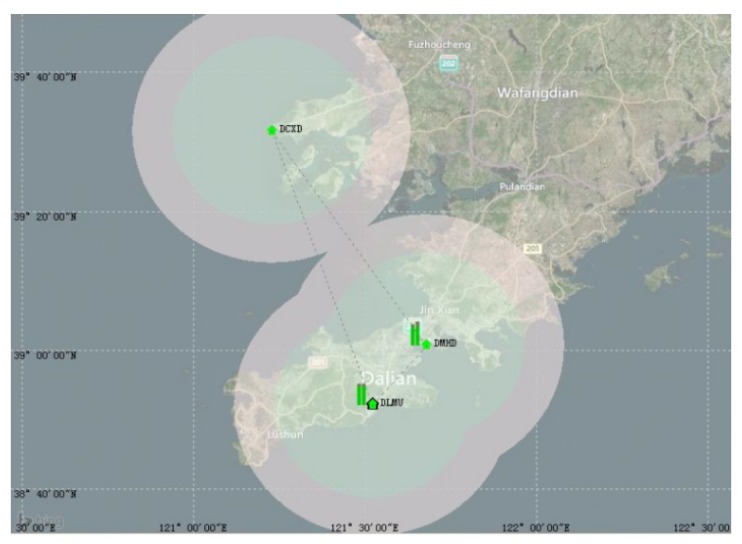
Distribution of DLMU-CORS network.

The DLMU-CORS network established on the coast of Dalian City is selected as the experimental basis for providing the differential corrections. The network has three reference stations, located at the Dalian Maritime University, Mianhua Island, and Changxing Island, respectively. Each CORS station passes its own observation data to CORS server which further distributes the data via the network. The distribution of the reference stations and the service range of each station are shown in Figure 6.

In this test, the AIS base station of Huangbaizui in Dalian is selected to broadcast the differential corrections. The test site was located in the water area near the AIS base station. The GDOP value in the test area was less than 1.5. The test ship was equipped with a network RTK mobile station, an AIS mobile station, and a GPS positioning module. The network RTK mobile station provides a millimeter-level high-precision positioning service. The AIS mobile station and GPS positioning module both use the RCB-4HGPS positioning chip (U-blox, Thalwil, Switzerland). The chip’s single-point positioning error is 6 m (2σ). The positioning of the RTK mobile station was taken to be the true value during the test. The AIS mobile station received the global navigation-satellite system broadcast binary messages broadcast by the AIS base station and performed differential positioning as per our method. The GPS positioning module performed single-point positioning. Positioning tests for all three methods were collected simultaneously and compared. Systematic accuracy was tested for two cases: static positioning and dynamic positioning.

### 5.2. Experimental Results

#### 5.2.1. Static Positioning

**Figure 7:**
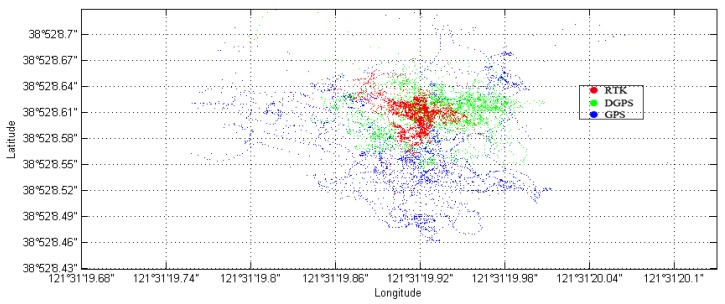
Real-time experimental results for static positioning.

The static experiment was tested on the ship at anchor. The test lasted 50 min with a sampling interval of one second. The positioning results are shown in Figure 7. The red marks are the results obtained by using network RTK high-precision positioning. It can be seen from the figure that the ship is not completely stationary due to the impact of the sea-air current and the wind direction, which caused the boat to move about its anchored position. The blue dots represent the positioning obtained using the existing single-point positioning. The green dots indicate the positioning result with the aid of the differential correction approach using broadcast binary messages. Clearly, the positioning error of this method is much smaller than that of single-point positioning and is closer to the value of RTK high-precision positioning.

**Figure 8:**
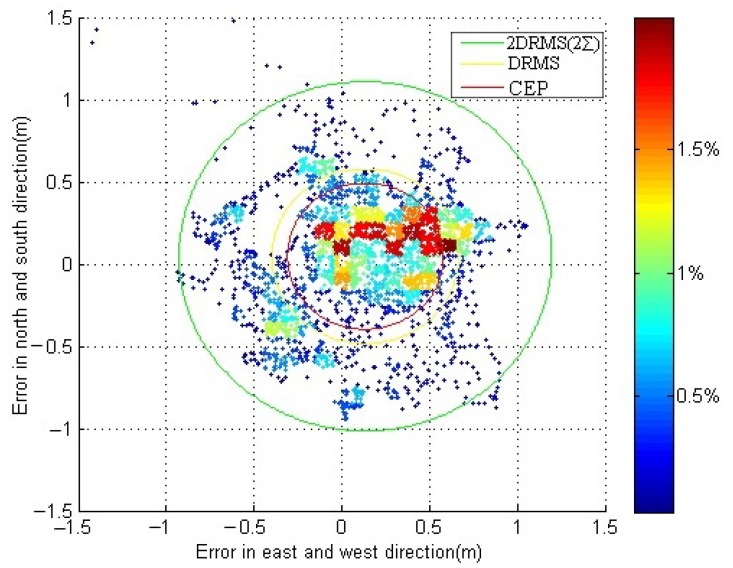
Scatter plot of the position errors for the static positioning experiment.

The static positioning errors are shown in Figure 8 in which the horizontal vertical axis represents the positioning error in longitude; the value of the mean $\mu_{x}$ and the standard errors $\sigma_{x}$ in that direction are 0.133 m and 0.38 m, respectively. The vertical axis represents the positioning error in latitude; the value of the mean $\mu_{y}$ and the standard errors $\sigma_{y}$ in that direction are 0.047 m and 0.37 m, respectively. The color of the points changes from red to blue, which indicates that the frequency of the points tends to reduce. The static positioning accuracy of is 1.06 m (2 DRMS).

#### 5.2.2. Dynamic Positioning Experiment

**Figure 9:**
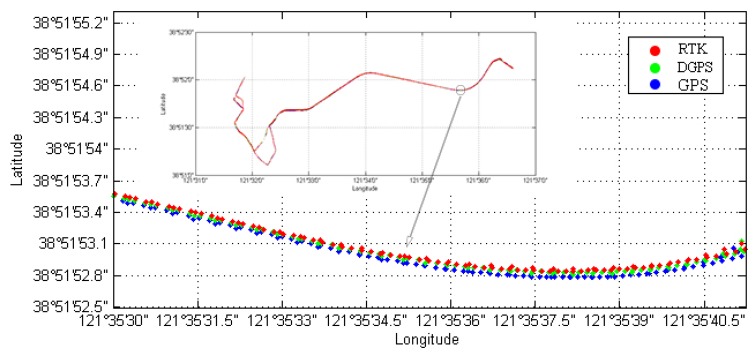
Vessel trajectory for the dynamic positioning experiment.

The dynamic test aimed to analyze the positioning accuracy of a traveling ship. The speed of the ship was about 20 knots. The test lasted 40 min and the sampling interval was 1 s. The dynamic positioning results are shown in Figure 9. Blue points indicate the results from single-point positioning and green points indicate the positioning results with the aid of differential corrections provided by our method. The result of high-precision RTK positioning is indicated by red points.

**Figure 10:**
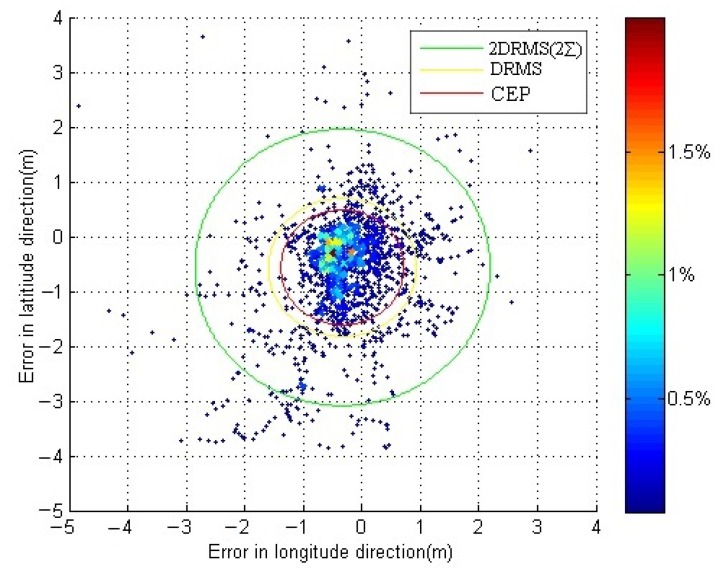
Scatter plot of the position errors for the dynamic positioning experiment.

The dynamic positioning errors are shown in Figure 10 in which the horizontal vertical axis represents the positioning error in longitude; the value of the mean $\mu_{x}$ and the standard errors $\sigma_{x}$ in that direction are −0.333 m and 0.82 m, respectively. The vertical axis represents the positioning error in latitude; the value of the mean $\mu_{y}$ and the standard errors $\sigma_{y}$ in that direction are −0.564 m and 0.96 m, respectively. The color of the points changes from red to blue, which indicates that the frequency of the points tends to reduce. The dynamic positioning accuracy of is 2.54 m (2 DRMS).

### 5.3. Discussion

Both the static and the dynamic positioning tests were performed in regions where the GDOP was less than 1.5. By analyzing the positioning results, we found that the accuracy of static positioning and dynamic positioning corrected by our approach (1.06 m (2DRMS) and 2.54 m (2DRMS), respectively) was better than the accuracy of the AIS single-point positioning system (2.15 m (2DRMS) and 5.52 m(2DRMS), respectively). Therefore, the differential service provided by this method has a significant improvement in terms of positioning accuracy.

## 6. Conclusions

In order to make up for the deficiency of differential corrections sources in current AIS and to improve the positioning accuracy of AIS mobile stations which do not have a DGPS receiver, a differential correction approach for AIS mobile stations based on the CORS network is proposed in this paper. AIS server derives real-time pseudo-range differential corrections from each reference station in CORS network and generates the corrections for AIS mobile stations. Then these differential corrections were transmitted by AIS base station to mobile stations using broadcast or addressed binary messages for positioning. After testing, it was found that the system could reliably provide differential corrections to AIS mobile stations. The system performed accurately for both static positioning and dynamic positioning, with accuracy values of 1.06 m (2DRMS) and 2.54 m (2DRMS), respectively. Compared with a single-point positioning method, the differential service effectively improved positioning accuracy. In addition, being based on the existing CORS network, investment in labor and financial resources are greatly reduced. Therefore, it is feasible that both cost-effectiveness and user-friendliness could contribute to a rapid deployment of this method.

## Figures and Tables

**Table 1:** Differential correction data parameter table.

Parameter	Number of bits	Description
*V*	index	–
Message type	6	Recommendation ITU-R M.823
Station ID	10	Recommendation ITU-R M.823 station identifier
Z count	13	Time value in 0.6 s (0-3 599.4)
Sequence number	3	Message sequence number (cyclic 0-7)
N	5	Number of DGNSS data words following the two word header, up to a maximum of 29
Health	3	Reference station health (specified in Recommendation ITU-R M.823)
DGNSS data word	N = 24	DGNSS message data words excluding parity
Number of bits	736	Assuming N = 29 (the maximum value)

**Table 2:** Notations.

Notation	Description
$M_{R}$	RTCM messages from each reference station
$\Delta \rho_{i}$	Differential corrections of each reference station
$C_{b}$	Coordinates of AIS base station
$\Delta \rho_{b}$	Differential corrections of AIS base station
$M_{G}$	AIS messages: Global navigation-satellite system broadcast binary message
$\rho_{m}$	Pseudoranges between satellites and the internal GPS receiver
$C_{m}$	Coordinates of AIS mobile station
$M_{p}$	AIS messages: Position report

**Table 3:** Parameters of the addressed binary message.

Parameter	Number of bits	Description
Message ID	6	Always 6
Repeat indicator	2	How many times a message has been repeated
Source ID	30	MMSI number of source station
Sequence number	2	0–3
Destination ID	30	MMSI number of destination station
Retransmit flag	1	0 = no retransmission = default; 1 = retransmitted
Spare	1	Should be zero. Reserved for future use
Binary data	Maximum 936	Application identifier and application data
